# Effects of SGLT2 inhibition on cardiac remodeling and heart failure development in aortic stenosis rats

**DOI:** 10.1186/s43556-026-00642-x

**Published:** 2026-10-28

**Authors:** Luana U. Pagan, Mariana J. Gomes, Mariana Gatto, Gustavo A. F. Mota, João Pedro G. Oliveira, Felipe C. Damatto, Lidiane M. Souza, Eder A. Rodrigues, Anna Clara C. Santos, Gabriela H. Tukiyama, Dijon H. S. Campos, Marcel Rodrigues Ferreira, Camila R. Correa, Gilson M. Murata, Bertha F. Polegato, Marina P. Okoshi, Katashi Okoshi

**Affiliations:** 1 Botucatu Medical School, UNESP, Botucatu, Brazil; 2 Department of Kinesiology and Sport Management, Texas A&M University, College Station, USA; 3 Laboratory of Medical Investigation, Clinic Medical Department, University of Sao Paulo Medical School, Sao Paulo, Brazil

**Keywords:** Cardiac failure, Left ventricular function, Empagliflozin, Transcriptome, Aorta stenosis

## Abstract

Aortic stenosis (AS) is increasingly prevalent in ageing populations, yet no pharmacological therapy has been shown to modify its progression to heart failure (HF). Sodium glucose cotransporter 2 (SGLT2) inhibitors improve outcomes in HF, but their effects in pressure overload-induced cardiac remodeling remain unclear. Here, we show that rats with long-term AS develop HF features, left ventricular dilation, and systolic and diastolic dysfunction, accompanied by increased oxidative stress, impaired antioxidant defenses, myocardial fibrosis, and transcriptional remodeling. Empagliflozin (10 mg/kg/day for 8 weeks) attenuated cardiac dysfunction and reduced HF features. At molecular level, empagliflozin prevented increase in lipid peroxidation, and decreased interstitial collagen deposition, while increasing glutathione peroxidase and matrix metalloproteinase-2 activity. Transcriptomic analysis identified 1,078 differentially expressed genes and revealed enrichment of pathways associated with extracellular matrix organization and signal transduction in AS. Empagliflozin predominantly regulated genes involved in cardiac contraction, mitochondrial membrane organization, apoptosis, and cell survival. Profibrotic and remodeling-associated genes were upregulated in AS, while empagliflozin induced a distinct transcriptional program associated with improved myocardial homeostasis. AS was associated with reduced activity of energy metabolism enzymes, which was not restored by empagliflozin. Collectively, these findings identify oxidative stress, extracellular matrix remodeling, and transcriptional reprogramming as mechanisms underlying cardiac dysfunction in AS. Empagliflozin mitigates cardiac remodeling and HF progression and modulates fibrosis and gene networks regulating cardiac function. These data highlight SGLT2 inhibition as a potential therapeutic strategy in pressure overload-induced heart failure.

## Introduction

Aortic stenosis (AS) is characterized by a progressive calcification of aortic valve leaflets and left ventricular (LV) outflow tract obstruction [1]. AS is the most common heart valve disease, and its prevalence is expected to increase as populations age [2]. Currently, AS affects 2% to 5% of individuals over 65 years of age and is associated with high morbidity and mortality [2, 3]. Severe symptomatic AS has a mortality rate of approximately 50% in 2 years [3].

Chronic pressure-overload induces genetic, molecular, cellular, and interstitial changes that clinically manifest as modifications in heart size, shape, and function. This process is known as cardiac remodeling [4]. AS is a classical experimental model that leads to substantial LV pressure overload with myocardial hypertrophy and fibrosis, evolving to LV diastolic and systolic dysfunction, heart failure, cardiac arrhythmia, and death [3, 5]. Other factors, besides myocardial hypertrophy and fibrosis, are involved in cardiac remodeling such as oxidative stress, inflammation, and energy deficit [6–8].

The gold standard treatment for severe AS is aortic valve replacement [9]. To date, no pharmacological therapies have shown improved AS natural history [9, 10]. Sodium-glucose cotransporter 2 (SGLT2) inhibitors were introduced to treat diabetes mellitus and heart failure in the last decade [11]. Their primary effect is to inhibit renal SGLT2 and reduce filtered glucose and sodium reabsorption [11, 12]. Surprisingly, SGLT2 inhibitors decreased mortality and the risk of hospitalization for heart failure in patients with Type 2 diabetes and high cardiovascular risk [12]. Their beneficial effects were later tested in different clinical scenarios; currently, SGLT2 inhibitors are approved for the treating patients with diabetes mellitus, heart failure with reduced or preserved ejection fraction, and chronic kidney disease [11, 13–16]**.**

The cardiovascular benefits observed in clinical trials with SGLT2 inhibitors were more than just decreased glycemia and blood volume. However, despite extensive research, the mechanisms involved in these benefits have not been fully established. Recent studies have shown that SGLT2 inhibitors attenuate myocardial oxidative stress, fibrosis, inflammation, and metabolic changes [17–21]. Empagliflozin also improved diastolic function in failing human hearts and murine heart failure models [22]. Therefore, SGLT2 inhibitors may have pleiotropic effects that can be beneficial in AS. In a recent retrospective multicenter observational study, Shah et al. [23] demonstrated that SGLT2 inhibition may slow non-severe AS progression.

In this study, we assessed the influence of chronic administration of SGLT2 inhibitor empagliflozin on cardiac remodeling in rats with long-term aortic stenosis. We also explored potential mechanisms that may underlie the empagliflozin effects in attenuating disease progression, such as myocardial fibrosis, oxidative stress, transcriptome changes, and the activity of enzymes involved in energy metabolism.

## Results

### Empagliflozin attenuates heart failure features frequency and mortality rate in AS rats

**Fig. 1:**
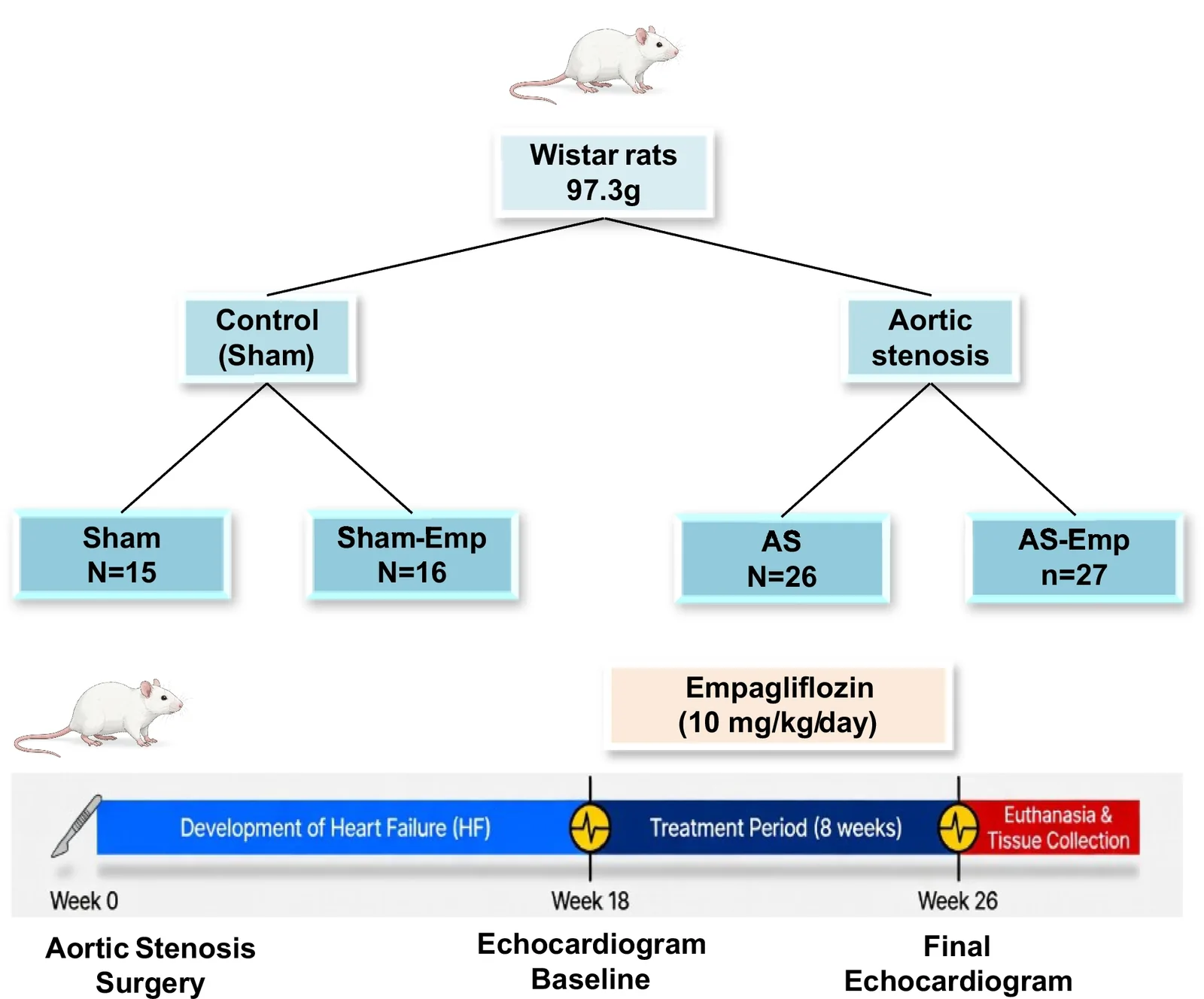
Schematic protocol timeline. Sham: control; Sham-Emp: control treated with empagliflozin; AS: aortic stenosis; AS-Emp: AS treated with empagliflozin; HF: heart failure

**Fig. 2:**
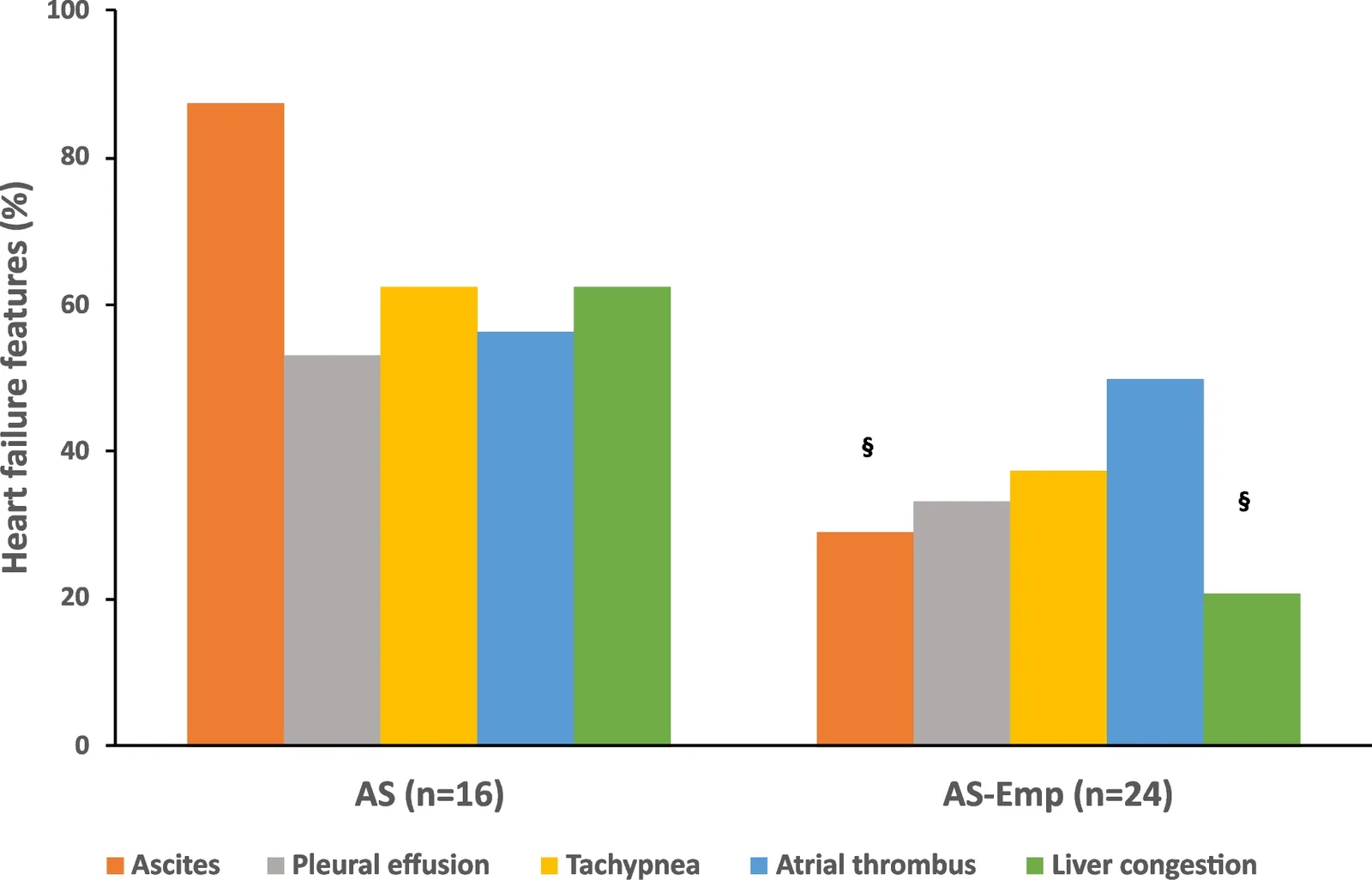
Heart failure feature frequencies. AS: aortic stenosis. AS-Emp: AS treated with empagliflozin. Goodman test; § *p* < 0.05 vs AS

All rats were subjected to AS or Sham surgery. A schematic protocol timeline is shown in Fig. 1. Perioperative mortality in AS rats was 29.2%. After starting treatment, 9 rats from AS and three from AS-Emp group died. Mortality was statistically lower in AS-Emp than AS (mortality rate: AS 33.3%; AS-Emp 11.1%; Goodman test; *p* < 0.05). No rats from Sham or Sham-Emp died. One rat from AS was excluded from the study because it developed extensive collateral circulation around the clip. The frequency of heart failure features for AS and AS-Emp groups is shown in Fig. 2. AS-Emp had a lower percentage of ascites and liver congestion than AS. Control rats did not have any features of heart failure.

Before starting treatment, body weight (BW) was lower in AS and AS-Emp than their respective controls (Sham 463.22 ± 53.03; Sham-Emp 457.14 ± 44.30; AS 418.25 ± 31.48; AS-Emp 418.23 ± 50.03 g; *p* < 0.05 AS and AS-Emp vs Sham and Sham-Emp, respectively). At the end of the experiment, BW was lower in Sham-Emp and AS than Sham (Table 1).

**Table 1:** Cardiac structural and left ventricular systolic and diastolic function

	**Sham (*n* = 14)**	**Sham-Emp (*n* = 14)**	**AS (*n* = 16)**	**AS-Emp (*n* = 17)**
BW (g)	509.07 ± 67.28	467.00 ± 44.40*	452.31 ± 29.94*	453.94 ± 35.68
HR (bpm)	258.36 ± 22.68	246.36 ± 32.17	245.50 ± 20.66	252.53 ± 32.28
LVDD (mm)	8.39 ± 0.57	8.25 ± 0.38	9.80 ± 0.53*	9.08 ± 0.87^#§^
LVSD (mm)	4.24 ± 0.68	4.19 ± 0.57	5.72 ± 0.74*	5.09 ± 1.08^#§^
LVPWT (mm)	1.37 (1.37–1.44)	1.41 (1.37–1.43)	2.30 (2.20–2.44)*	2.16 (2.12–2.26)^#^
AO (mm)	4.01 (3.99–4.22)	4.10 (3.94–4.17)	4.10 (3.94–4.15)	3.94 (3.94–4.13)
LA (mm)	5.68 ± 0.40	5.64 ± 0.32	9.67 ± 1.00*	8.97 ± 1.36^#§^
LA/AO	1.39 ± 0.09	1.38 ± 0.09	2.39 ± 0.26*	2.23 ± 0.36^#^
LVDD/BW (mm/kg)	16.72 ± 2.36	17.81 ± 1.79	21.84 ± 1.77*	20.14 ± 1.42^#§^
LA/BW (mm/kg)	11.30 ± 1.35	12.12 ± 1.05	21.44 ± 2.34*	19.92 ± 3.41^#^
LVM (g)	0.85 ± 0.11	0.82 ± 0.07	2.19 ± 0.27*	1.76 ± 0.34^#§^
LVMI (g/kg)	1.68 ± 0.25	1.77 ± 0.24	4.86 ± 0.70*	3.90 ± 0.76^#§^
RWT	0.33 ± 0.03	0.34 ± 0.02	0.48 ± 0.03*	0.48 ± 0.04^#^
EFS (%)	49.72 ± 5.77	49.34 ± 5.33	41.78 ± 5.30*	44.40 ± 7.77^#^
PWSV (mm/s)	41.46 ± 5.33	39.43 ± 4.17	26.92 ± 3.32*	27.23 ± 4.61^#^
Tei index	0.48 ± 0.06	0.48 ± 0.07	0.54 ± 0.09	0.50 ± 0.11
S’ (cm/s)	3.42 ± 0.31	3.12 ± 0.44*	2.42 ± 0.34*	2.68 ± 0.30^#§^
Mitral E (cm/s)	81 (72—87)	77 (72—81)	133 (123–137)*	118 (103–135)^#^
Mitral A (cm/s)	47.5 (42.5—55.5)	51.5 (39.3—58.3)	17.0 (15.3—24.8)*	17.0 (15.0—34.0)^#^
E/A	1.56 (1.45—1.99)	1.59 (1.31—1.91)	7.64 (5.39—9.10)*	6.77 (3.03—9.33)^#^
IVRT (ms)	28.57 ± 2.28	29.79 ± 3.04	18.94 ± 2.59*	22.94 ± 5.02^#§^
E’ (average, cm/s)	4.11 ± 0.40	3.89 ± 0.58	3.83 ± 0.54	3.89 ± 0.81
E/E’	19.60 ± 2.76	19.86 ± 2.92	35.00 ± 7.15*	30.67 ± 4.84^#§^
A’ (average, cm/s)	3.47 ± 0.45	3.63 ± 0.80	2.45 ± 0.41*	2.98 ± 0.75^#§^

Data are means ± SD or medians and percentiles. *Sham* control, *Sham-Emp* control treated with empagliflozin, *AS* aortic stenosis, *AS-Emp* AS treated with empagliflozin, *BW* body weight, *HR* heart rate, *LVDD and LVSD* left ventricular (LV) diastolic and systolic diameters, respectively, *LVPWT* LV posterior wall thickness, *AO* aorta diameter, *LA* left atrial diameter, *LVM* LV mass, *LVMI* LVM index, *RWT* relative wall thickness, *EFS* LV endocardial fractional shortening, *PWSV* LV posterior wall shortening velocity, *S'* mitral annular systolic velocity, *TDI* averaged lateral and septal wall velocities, *E/A* ratio of early (E) to late (A) diastolic mitral inflow velocities, *IVRT* isovolumic relaxation time, *E'* TDI of early diastolic velocity of mitral annulus, *A'* TDI of late mitral annular velocity. ANOVA and Tukey or Kruskal–Wallis and Dunn test

^*^p < 0.05 vs Sham

^#^*p* < 0.05 vs Sham-Emp

^§^*p* < 0.05 vs AS

In summary, long-term AS-induced mortality was elevated; surviving rats had a high frequency of pathological heart failure features. Mortality and heart failure development were attenuated by empagliflozin.

### SGLT2 inhibition attenuates cardiac structural changes and LV dysfunction

In this section, we evaluated cardiac remodeling by transthoracic echocardiogram performed under light anesthesia. Cardiac structure and left ventricular (LV) function were assessed before initiating the treatment (data not shown) and at the end of the experiment by transthoracic echocardiography. Rats with poor acoustic window were excluded from the echocardiographic analysis, which resulted in a smaller sample size for image parameters than anatomical data. No differences were observed between AS-Emp and AS in echocardiogram performed before treatment (data not shown). Echocardiographic evaluation of cardiac structures and LV function at the end of the experiment is presented in Table 1. AS and AS-Emp groups had greater LVDD, LVSD, LVPWT, LA, LA/AO ratio, LVDD/BW, LA/BW, LVM, LVMI and relative wall thickness than their respective controls. AS-Emp presented lower LVDD, LVSD, LA, LVDD/BW, LVM and LVMI values ​than AS.

The systolic parameters endocardial fractional shortening, posterior wall shortening velocity, and S’ were lower in AS and AS-Emp than their respective controls. Tei index did not differ between groups. AS-Emp had higher S’ than AS. The diastolic variables E wave, E/A ratio, and E/E' were higher, and A wave, IVRT, and lateral, septal, and average A' lower in AS-Emp and AS than their respective controls. AS-Emp had higher IVRT, average and septal A' wave, and lower E/E' than AS.

In summary, aortic stenosis groups had left chambers dilation, LV concentric hypertrophy, characterized by the increased relative wall thickness, and systolic and diastolic dysfunction. Empagliflozin attenuated left chambers dilation, LV hypertrophy, and LV systolic and diastolic dysfunction.

### Empagliflozin attenuates myocardial fibrosis

To address changes in myocardial hypertrophy and fibrosis, we assessed anatomical variables, myocyte diameter and interstitial collagen fraction. Table 2 shows anatomical parameters. LV, right ventricle (RV), and atria weights in absolute or normalized to body weight values, and liver wet/dry ratio were higher in AS and AS-Emp than their controls. AS-Emp had lower LV wet-to-dry weight ratio than AS.

**Table 2:** Cardiac anatomical data and heart, lung, and liver wet-to-dry weight ratio

	**Sham (*n* = 15)**	**Sham-Emp (*n* = 16)**	**AS (*n* = 16)**	**AS-Emp (*n* = 24)**
LV (g)	0.96 ± 0.11	0.92 ± 0.09	1.64 ± 0.20*	1.58 ± 0.14^#^
LV/BW (g/kg)	1.97 ± 0.16	2.05 ± 0.12	3.80 ± 0.41*	3.79 ± 0.39^#^
RV (g)	0.23 (0.21–0.26)	0.22 (0.20–0.25)	0.51 (0.44–0.57)*	0.45 (0.36–0.50)^#^
RV/BW (g/kg)	0.49 (0.44–0.55)	0.49 (0.45–0.55)	1.15 (1.08–1.34)*	1.10 (0.91–1.24)^#^
Atria (g)	0.10 (0.09–0.12)	0.10 (0.09–0.12)	0.38 (0.28–0.44)*	0.32 (0.25–0.39)^#^
Atria/BW (g/kg)	0.21 (0.19–0.24)	0.22 (0.21–0.24)	0.91 (0.65–0.99)*	0.77 (0.63–1.03)^#^
LV wet/dry	3.81 ± 0.32	3.90 ± 0.38	4.38 ± 0.16*	4.17 ± 0.33^#§^
RV wet/dry	4.16 (4.06–4.25)	4.15 (4.08–4.25)	4.54 (4.49–4.60)*	4.47 (4.17–4.56)^#^
Atria wet/dry	4.28 ± 0.48	4.13 ± 0.40	4.73 ± 0.30*	4.66 ± 0.39^#^
Lung wet/dry	4.56 (4.45–4.77)	4.62 (4.50–4.66)	4.73 (4.58–4.94)	4.66 (4.45–4.78)
Liver wet/dry	3.05 (3.00–3.11)	3.08 (2.99–3.10)	3.18 (3.07–3.28)*	3.16 (3.11–3.25)^#^

Data are means ± SD or medians and percentiles. *Sham* control, *Sham-Emp* control treated with empagliflozin, *AS* aortic stenosis, *AS-Emp* AS treated with empagliflozin, *BW* body weight, *LV* left ventricle weight, *RV* right ventricle weight, *wet/dry* wet-to-dry tissue weight ratio. ANOVA with Tukey or Kruskal–Wallis with Dunn test

^*^p < 0.05 vs Sham

^#^*p* < 0.05 vs Sham-Emp

^§^*p* < 0.05 vs AS

**Fig. 3:**
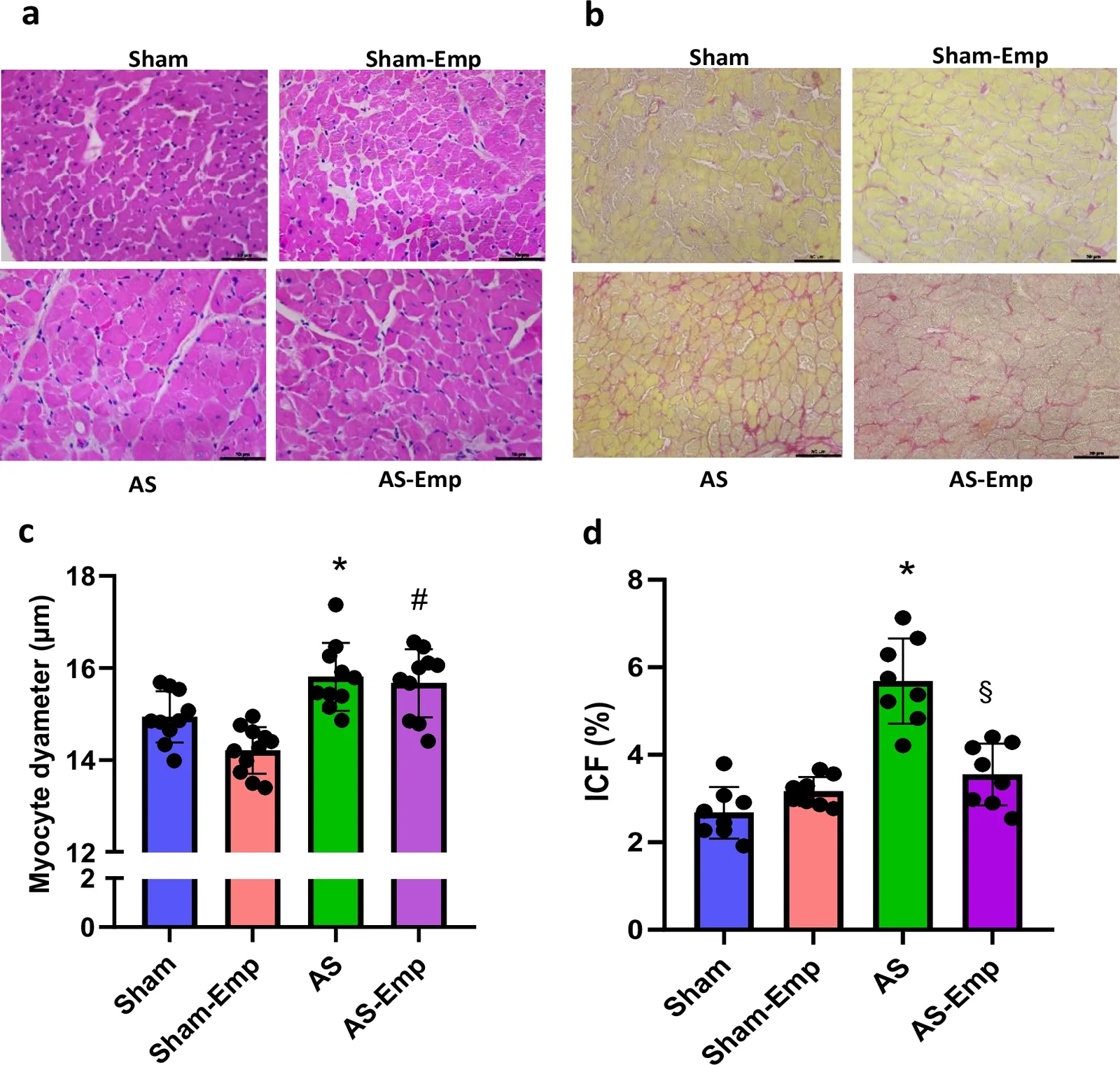
Histology evaluation shows myocyte hypertrophy in both AS and AS-Emp groups and ICF reduction by empagliflozin in AS-Emp. **a** representative histological sections of hematoxylin and eosin-stained left ventricular (LV) myocardium; **b** representative histological sections of picrosirius red-stained LV myocardium; **c** myocyte diameter; **d** interstitial collagen fraction (ICF). Sham: control; Sham-Emp: control treated with empagliflozin; AS: aortic stenosis; AS-Emp: AS treated with empagliflozin. ANOVA and Tukey test; * *p* < 0.05 vs Sham; # *p* < 0.05 vs Sham-Emp; § *p* < 0.05 vs AS; *n* = 10 per group

Myocyte diameter and interstitial collagen fraction were assessed through optical microscopy. Representative hematoxylin and eosin and picrosirius red-stained histological sections of LV myocardium are shown in Fig. 3. Myocyte diameter was higher in AS and AS-Emp than their respective controls. Interstitial collagen fraction was higher in AS than control and AS-Emp groups (Fig. 3). In summary, the increased water content in organs suggests interstitial fluid overload following heart failure development, which was attenuated in the LV of AS-Emp compared to AS group. Myocardial fibrosis was attenuated by empagliflozin.

### SGLT2 inhibition restores glutathione peroxidase and increases intermediate metalloproteinase-2 activity

In this section, we evaluated myocardial and serum oxidative stress markers, metalloproteinase-2 activity, and energy metabolism enzyme activities. These variables are often altered in cardiac remodeling [4, 8]. Seven rats per group were randomly selected for oxidative stress evaluation and metalloproteinase-2 activity and 8 rats per group for energy metabolism enzyme activities (Table 3). All assays were performed in triplicate, including samples and standard curves.

**Table 3:** Myocardial and serum oxidative stress markers, metalloproteinase (MMP)−2 quantification, and energy metabolism enzyme activities

	**Sham (*n* = 7–8)**	**Sham-Emp (*n* = 7–8)**	**AS (*n* = 7–8)**	**AS-Emp (*n* = 7–8)**
Lipid hydroperoxide (nmol/g)	121.08 ± 25.91	140.35 ± 21.96	188.65 ± 42.15*	158.65 ± 30.47
Catalase (µmol/g)	29.43 ± 2.79	27.91 ± 4.75	21.24 ± 2.60*	23.75 ± 2.45^#^
SOD (nmol/mg)	7.81 ± 1.95	7.03 ± 0.96	8.21 ± 1.15	7.51 ± 1.73
GSH-Px (nmol/g)	31.90 ± 4.52	29.54 ± 3.72	21.98 ± 3.09*	28.37 ± 2.47^§^
Carbonilation (nmol/mg protein)	152.01 ± 30.31	160.23 ± 17.90	166.12 ± 20.21	168.11 ± 21.40
MDA (nmol/mg protein)	1.53 ± 0.50	1.51 ± 0.27	1.55 ± 0.16	1.67 ± 0.30
Superior MMP-2	1.25 (1.00–1.76)	1.12 (0.79–2.49)	1.74 (1.07–2.76)	3.15 (2.54–6.25)^#^
Intermediate MMP-2	1.39 ± 0.28	1.23 ± 0.42	1.54 ± 0.42	1.97 ± 0.42^#§^
Inferior MMP-2	0.75 (0.67–1.59)	0.50 (0.16–1.25)	1.84 (0.38–3.26)	1.75 (0.82–3.35)
HK	43.45 ± 7.96	48.66 ± 9.33	43.23 ± 6.67	49.92 ± 4.47
PFK	61.66 ± 6.48	67.81 ± 6.60	51.53 ± 5.86*	51.36 ± 5.80^#^
PK	1112.16 ± 62.06	1043.48 ± 96.16	1121.19 ± 99.97	1114.68 ± 100.51
LDH	186.14 ± 17.66	187.54 ± 24.85	183.24 ± 18.96	209.61 ± 23.38
CK	198.51 ± 10.46	198.17 ± 17.42	133.50 ± 20.17*	123.48 ± 8.94^#^
CS	182.65 ± 10.71	174.6 ± 26.36	136.42 ± 15.09*	128.51 ± 15.93^#^
BHADH	71.18 ± 9.89	66.61 ± 13.13	28.43 ± 8.51*	19.59 ± 8.69^#^
CPT1	2.42 ± 0.43	2.45 ± 0.83	2.93 ± 0.75	2.86 ± 0.38

Data are means ± SD or medians and percentiles. *Sham* control, *Sham-Emp* control treated with empagliflozin, *AS* aortic stenosis, *AS-Emp* AS treated with empagliflozin, *SOD* superoxide dismutase, *GSH-Px* glutathione peroxidase, *Carbonilation* protein carbonilation, *MDA* malondialdehyde, *HK* hexokinase, *PFK* phosphofructokinase, *PK* pyruvate kinase, *LDH* lactate dehydrogenase, *CK* creatine kinase, *CS* citrate synthase, *BHADH* β-hydroxyacyl-CoA dehydrogenase, *CPT1* carnitine palmitoyltransferase 1. ANOVA and Tukey or Kruskal–Wallis and Dunn test

^*^p < 0.05 vs Sham

^#^*p* < 0.05 vs Sham-Emp

^§^*p* < 0.05 vs AS

AS had higher lipid hydroperoxide concentration and lower catalase and glutathione peroxidase activities than Sham. AS-Emp presented reduced catalase activity than Sham-Emp and increased glutathione peroxidase than AS. Superoxide dismutase did not differ between groups. AS-Emp had a higher superior metalloproteinase-2 band than Sham-Emp, and higher intermediate metalloproteinase-2 band than Sham-Emp and AS. AS and AS-Emp had lower activity of phosphofructokinase, creatine kinase, citrate synthase and β-hydroxyacyl-CoA dehydrogenase compared to their respective controls. Hexokinase, pyruvate kinase, lactate dehydrogenase and carnitine palmitoyl transferase 1 did not differ between groups (Table 3).

In summary, AS group had higher oxidative stress and lower antioxidant capacity than Sham. The empagliflozin normalized the activity of glutathione peroxidase and increased intermediate MMP-2. Activity of enzymes involved in energy metabolism was changed by AS and not modulated by the treatment.

### Myocardial transcriptome

**Fig. 4:**
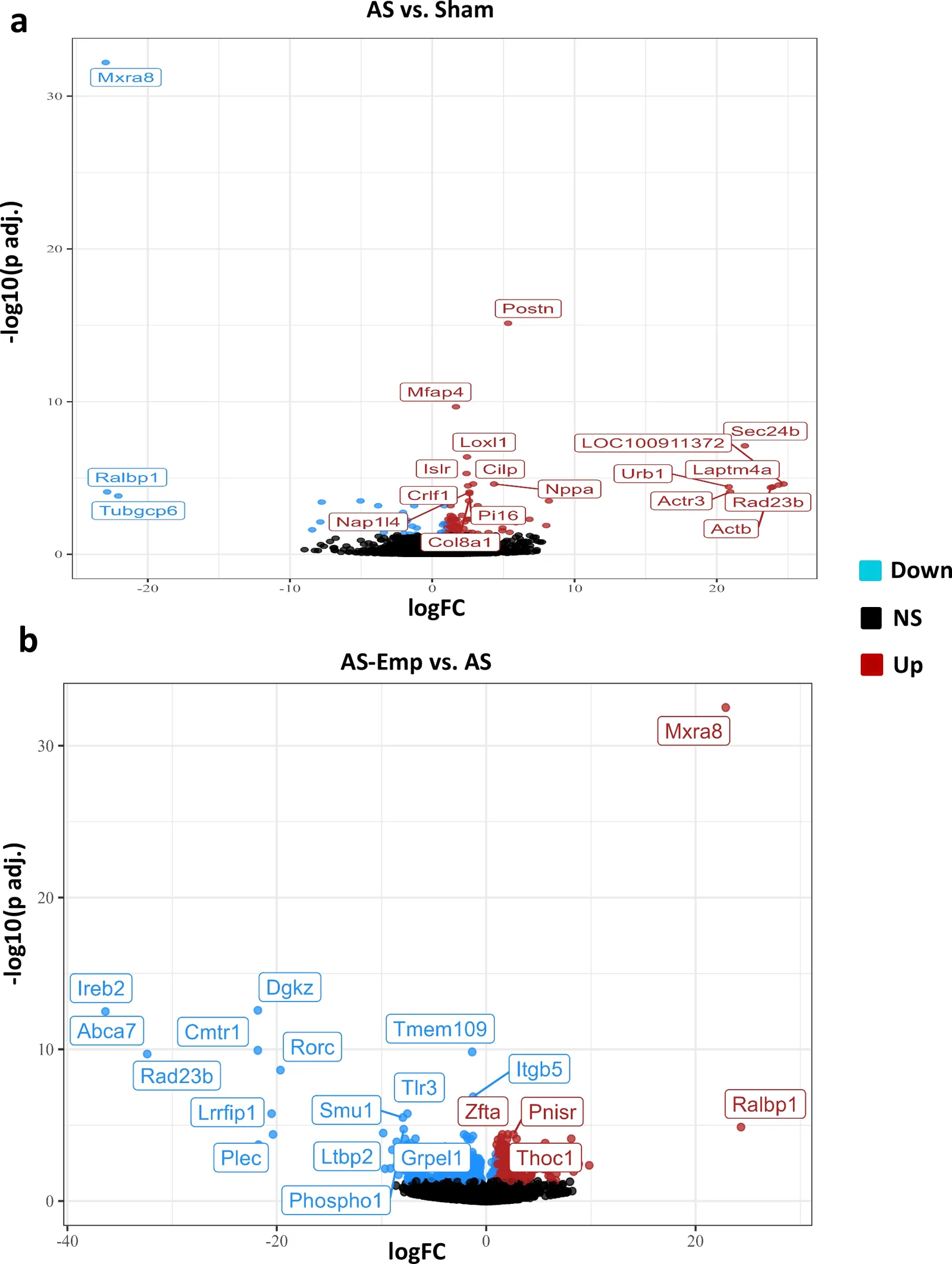
Volcano plot of cardiac transcripts analyzed using next-generation sequencing considering the comparisons AS vs Sham (**a**) and AS-Emp vs AS (**b**). Significantly downregulated genes (Down) are shown in blue and significantly upregulated genes (Up) in red; non-significantly altered genes (NS) are shown in black

We next evaluated myocardial global transcriptional profile by using next-generation sequencing (NGS) considering the comparisons AS vs Sham and AS-Emp vs AS. A total of 1,078 differentially expressed genes were identified, including 662 upregulated genes (69 in AS vs Sham; 593 in AS-Emp vs AS) and 416 downregulated genes (28 in AS vs Sham; 388 in AS-Emp vs AS). However, significant differences in gene abundance were observed only for the genes highlighted in Fig. 4. Comparison of AS and Sham identified *Loxl1, Mfap4, Postn, Col8a1, Pi16,* and *Nppa* among the main upregulated genes, while *Mxra8, Ralbp1,* and *Tubgcp6* were among the main downregulated genes. Comparison of AS-Emp and AS identified *Mxra8, Zfta, Pnisr, Thoc1,* and *Ralbp1* among the main upregulated genes, while *Itgb5, Ltbp2, Tmem109, Plec,* and *Rorc* were among the main downregulated genes.

**Fig. 5:**
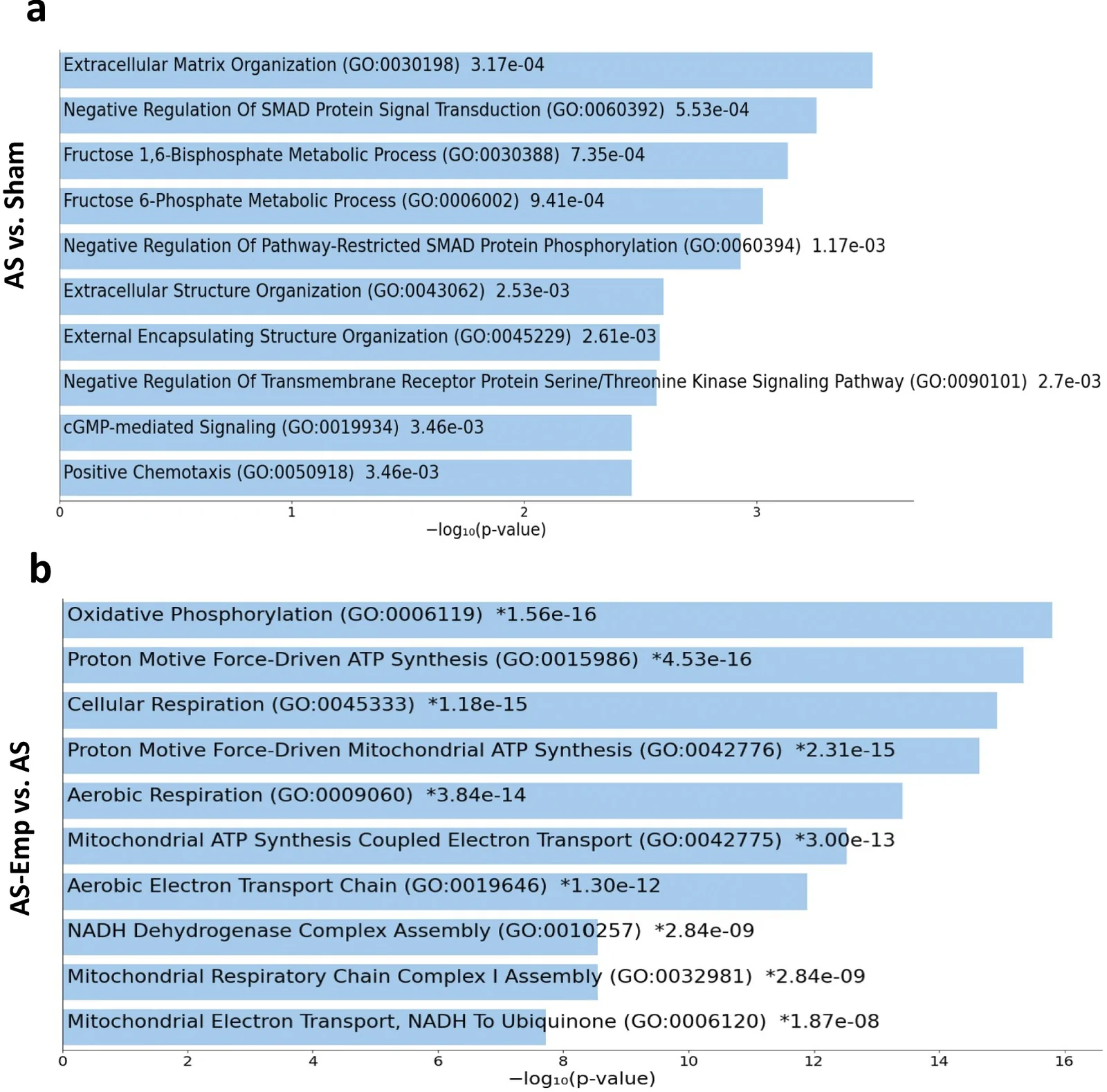
Bar graph of the top enriched terms from the GO_Biological_Process_2023 gene set library. Comparisons between the groups aortic stenosis (AS) vs. Sham (**a**) and AS treated with empagliflozin (AS-Emp) vs. AS (**b**)

Figure 5 summarizes the Gene Ontology (GO) enrichment analysis using the GO_Biological_Process_2023 library in Enrichr. The top 10 most significantly enriched biological processes are ranked according to -log10 (*p*-value), with the corresponding *p*-values indicated for each term. All displayed biological processes were significantly enriched, with the highest-ranked term showing the strongest enrichment.

In summary, the differentially expressed genes are mainly involved in biological processes related to apoptosis, mitochondrial organization, and extracellular matrix collagen remodeling, and may contribute to the pathophysiological mechanisms underlying aortic stenosis-induced heart failure and molecular responses associated with empagliflozin treatment.

## Discussion

In this study, we showed for the first time that treatment with the SGLT2 inhibitor empagliflozin for 8 weeks attenuated cardiac remodeling in rats with long-term aortic stenosis.

Ascending AS in rodents has often been used to study chronic pressure overload-induced cardiac remodeling. In this model, a silver clip is placed around the ascending aorta in young rodents. Immediately after clipping, the aorta diameter is preserved. AS and LV hypertrophy develop progressively as the animals grow. Rats develop LV hypertrophy 1 month later, diastolic and systolic dysfunction approximately 18‐28 weeks later, and finally clinical heart failure [5]. Therefore, the model is similar to that observed in human chronic pressure overload. The high frequency of heart failure features and mortality in the untreated rats show that AS was severe at the end of the experiment.

Treatment was initiated 18 weeks post-surgery, when AS rats typically begin to present LV dysfunction [24]. At this time, no rat had tachypnea/labored respiration. Following treatment, the AS-Emp group had lower mortality rate and reduced frequency of heart failure features such as ascites and hepatic congestion than AS.

Prior to empagliflozin administration, echocardiography was performed to ensure uniform distribution between study groups (data not shown). A follow-up echocardiogram was conducted at the end of the experimental period. Empagliflozin did not modulate any echocardiographic parameters in the Sham rats, except for lower S’ in Sham-Emp vs Sham. AS was characterized by left chambers dilation and LV concentric hypertrophy with systolic and diastolic dysfunction. Empagliflozin attenuated left chambers dilation and LV hypertrophy. Systolic and diastolic dysfunction was also attenuated by the treatment as AS-Emp had higher S’ and IVRT and lower E/E’ than AS. The fact that 18 weeks after inducing AS and before initiating treatment, no differences between AS-Emp and AS were observed suggests that the effects of empagliflozin were not only hemodynamic, but attenuation in cardiac remodeling in long-term AS rats.

Byrne et al. [25] previously demonstrated in short-term AS mice with reduced ejection fraction that SGLT2 inhibition prevented a progressive decrease in LV function without changing LV size. Clinical studies have suggested that SGLT2 inhibitors may have a beneficial effect in diastolic function in cardiovascular disease [26–28].

Post-mortem anatomical variables and myocyte diameters analysis confirmed echocardiographic findings of cardiac hypertrophy in AS. However, no differences between AS-Emp and AS were observed. One direct effect of SGLT2 inhibition is to induce natriuresis and diuresis [11]. Even so, BW did not differ between AS-Emp and AS.

Previous clinical trials have shown that SGLT2 inhibition significantly reduces the risk of cardiovascular death and heart failure hospitalization in patients with diabetes mellitus, heart failure, or both. However, the action mechanisms of this drug class are still not fully understood with several hypotheses having been raised to justify the effects of SGLT2 inhibitors [29–32].

Oxidative stress is caused by an imbalance in reactive oxygen species (ROS) production and antioxidant capacity and is strongly implicated in heart failure development. The increased production of free radicals during metabolic processes activates antioxidant defense mechanisms, including superoxide dismutase (SOD), catalase (CAT), and glutathione peroxidase (GSH-Px), as well as non-enzymatic systems. Under pathophysiological conditions, endogenous ROS accumulate in cardiac mitochondria, impairing mitochondrial function. Prolonged chronic exposure to ROS further induces apoptosis, necrosis, and fibrosis [33]. This study showed higher oxidative stress in AS than Sham, characterized by higher lipid hydroperoxide, the oxidative stress marker. Values for this marker in AS-Emp fell between Sham-Emp and AS values without significant differences, suggesting that empagliflozin may have attenuated oxidative stress. Furthermore, glutathione peroxidase antioxidant activity was higher in AS-Emp than AS. These findings suggest a potential protective effect from empagliflozin against oxidative stress in the myocardium of AS rats.

Interstitial fibrosis is an important component of pressure-overload-induced heart failure [34]; our study confirmed this by the higher interstitial collagen fraction in AS vs. Sham. Interestingly, this fraction was lower in AS-Emp vs. AS. The higher MMP-2 activity in AS-Emp vs. AS may have contributed to this reduced interstitial collagen fraction. MMPs degrade extracellular matrix proteins with different degrees of specificity [35]. Several MMPs are present in the heart, including collagenases, which initiate extracellular matrix degradation, and gelatinases, which act in the subsequent digestion of collagen fragments [35]. Other factors may have contributed to the reduced myocardial fibrosis, such as the attenuation of oxidative stress in AS-Emp. Furthermore, several mechanisms have been implicated in the antifibrotic effect of SGLT2 inhibitors. They suppress profibrotic signaling in cardiac fibroblasts and improve mitochondrial function and autophagic flux in myocytes, thus reducing oxidative stress. Immune modulation may also play a role in preventing myocardial fibrosis [36].

Abnormalities in myocardial energy metabolism precede and contribute to the progression of heart failure [37]. Energy metabolism data showed that phosphofructokinase, creatine kinase, citrate synthase and β-hydroxyacyl-CoA dehydrogenase activity was lower in both AS groups. Decrease in β-hydroxyacyl-CoA dehydrogenase activity shows worsening lipid oxidation which can affect the acetyl CoA production in the citric acid cycle. Citrate synthase catalyzes the first citric acid cycle reaction which forms citrate from acetyl-CoA and oxaloacetate. The decrease in citrate synthase impairs ATP production through this pathway. Glycolysis also appears to be impaired, as phosphofructokinase activity was reduced in both AS groups. Several authors consider that a beneficial effect of SGLT2 inhibition is modulation of myocardial metabolism [38]. Although we have not observed empagliflozin-induced changes in the activity of energy metabolism enzymes, additional studies are needed to evaluate myocardial metabolism.

We finally evaluated myocardial transcriptome. It was surprising to observe that a higher number of genes were differentially expressed in AS-Emp vs AS than in AS vs Sham. A positive regulation of *Loxl1, Mfap4, Postn,* and *Col8a1* was observed between AS and Sham. These genes are involved in collagen cross-linking, elastic fiber and fibrillin formation, tissue regeneration, and extracellular matrix composition [39]. Among essential biological processes influenced by the differentially expressed genes, we highlight the regulation of SMAD proteins associated with the transforming growth factor beta (TGF-β) pathway and fibrosis, extracellular matrix organization and glycolytic metabolism [40]. In accordance, we observed alteration in the glycolytic pathway through impaired phosphofructokinase activity. The increase in extracellular matrix-related biological processes may have contributed to the higher interstitial collagen fraction in AS.

The main statistically downregulated genes, *Ltbp2, Tmem109, Plec,* and *Rorc,* in AS-Emp vs AS are associated with the TGF-β pathway, autophagy, apoptotic cleavage, and cytokine production [41]. Increased *Ltbp2* expression was reported in animal and human heart failure [42, 43]. Therefore, *Ltbp2* downregulation in our study can be involved in the protective effects of empagliflozin. Regarding the upregulated genes, *Thoc1, Pnisr,* and *Zfta* are related to RNA splicing, RNA binding, and DNA regulation, and *Ralbp1* plays several cell roles, including oxidative stress response and apoptosis regulation [44]. The main biological processes influenced by the differentially expressed genes are related to mitochondrial activity, energy production, and cell respiration. Therefore, our data show that empagliflozin regulates genes important for cardiomyocyte homeostasis. Nonetheless, *Mxra8*, another upregulated gene, encodes the matrix remodeling-associated protein 8, involved in extracellular matrix remodeling and the interaction between cells and the surrounding matrix [45]. Although the upregulated *Mxra8* suggests the potential for empagliflozin to exacerbate cardiac fibrosis, this was not observed in this study [46].

In summary, our data show that empagliflozin attenuated oxidative stress and increased glutathione peroxidase antioxidant activity, and regulated genes involved in cardiac contraction, mitochondrial membrane organization, apoptosis, and cell survival. Some consequences of attenuated oxidative stress were observed in this study; these were myocardial fibrosis attenuation and LV function improvement. Myocardial fibrosis is strongly related to diastolic dysfunction. Therefore, as expected, diastolic dysfunction was attenuated by empagliflozin. Probably, due to improved LV function, the pathological features of heart failure were also attenuated in AS-Emp. There is concern in clinical practice that SGLT2 inhibitors, by promoting diuresis and reducing blood pressure, could impair AS hemodynamics [30]. Our study showed empagliflozin did not increase mortality. Finally, empagliflozin did not modulate the energy metabolism enzyme activities. Changes in energy metabolism have been strongly hypothesized as fundamental to the clinical benefits of SGLT2 inhibitors [47].

The exclusion of female rats was a limitation in this study. Sex-specific factors, including the influence of ovarian hormones, may impact ventricular remodeling in response to pressure overload and empagliflozin treatment and the translational potential of this study.

## Conclusion

Empagliflozin reduces the frequency of heart failure features and attenuates cardiac remodeling and left ventricular dysfunction in long-term aortic stenosis rats. Cardiac remodeling improvement is combined with increased glutathione peroxidase and MMP-2 activities, attenuated myocardial fibrosis, and modulation of gene networks regulating cardiac function. These data highlight SGLT2 inhibition as a potential therapeutic strategy in pressure overload-induced heart failure.

## Materials and methods

### Experimental animals and study design

Male Wistar rats were obtained at 30 days of age from the Central Animal House of Botucatu Medical School, São Paulo State University (UNESP), Brazil. Their initial body weight was 97.3 ± 8.4 g, and the housing temperature was maintained at 23 °C. The institutional Animal Research Ethics Committee approved the study under protocol 1271/2018; procedures followed the guidelines of the Brazilian College for Animal Experimentation.

Surgery was performed in 119 animals. For aortic stenosis (AS) induction, ketamine hydrochloride (50 mg/kg) and xylazine hydrochloride (10 mg/kg) were administered intramuscularly. A median thoracotomy provided access to the ascending aorta, which was dissected before a silver clip with an internal diameter of 0.6 mm was positioned approximately 3 mm distal to the aortic root [5]. Manual ventilation with 100% oxygen was provided during the operation. Sham surgery involved the same steps except aortic clipping. Postoperative analgesia comprised dipyrone sodium in drinking water (300 mg/kg) and intraperitoneal tramadol (5 mg/kg every 8 h for 72 h).

At postoperative week 18, surviving animals underwent transthoracic echocardiography to characterize cardiac injury before allocation to four groups: Sham (*n* = 15), Sham-Emp (*n* = 16), AS (*n* = 26), and AS-Emp (*n* = 27). Sham-Emp and AS-Emp received empagliflozin (Boehringer-Ingelheim, Ingelheim am Rhein, Germany). The drug was incorporated into chow at a dose of 10 mg/kg/day for eight weeks. Echocardiography was repeated after this treatment period, and euthanasia performed the following day. Figure 1 summarizes the experimental schedule.

### Echocardiographic assessment

Echocardiographic recordings were acquired and measured in accordance to the previously reported procedures [48–50]. Imaging used a Vivid S6 system (General Electric Medical Systems, Tirat Carmel, Israel) with a multifrequency transducer operating at 5–11.5 MHz. Treatment allocation was concealed from the examiner (K.O.). Anesthesia consisted of ketamine at 50 mg/kg and xylazine at 0.5 mg/kg administered intraperitoneally. Two-dimensional images guided the placement of M-mode recordings. The left ventricular (LV) short-axis recording plane was positioned immediately below the mitral leaflet tips; additional recordings were obtained at the aortic valve and left atrium. Tracings were printed with a Sony UP-890MD thermal printer using a sweep speed of 100 mm/s. Each reported measurement represented the average of at least five cardiac cycles. Structural measurements comprised LV cavity dimensions at diastole (LVDD) and systole (LVSD), left atrial (LA) and aortic (AO) diameters, and diastolic thickness of the LV posterior wall (LVPWT) and septum (LVSWT). The derived indices were LV mass, LVM = 1.04 × [(LVDD + LVPWT + LVSWT)^3^ − LVDD^3^], and relative wall thickness, RWT = (2 × LVPWT)/LVDD. Functional assessment included endocardial fractional shortening (EFS), posterior wall shortening velocity (PWSV), isovolumetric relaxation time (IVRT), and the Tei myocardial performance index. Mitral inflow recordings yielded early (E) and late (A) velocities and the E/A ratio. Systolic (S′), early diastolic (E′), and late diastolic (A′) annular velocities were obtained by tissue Doppler; E/E′ was then calculated.

### Anatomical assessment and heart failure features

Tachypnea or labored breathing was recorded as a clinical feature suggestive of heart failure. At necropsy, pleural fluid accumulation, atrial thrombi, ascites, and hepatic congestion were assessed as evidence of cardiac decompensation [51]. Following thoracotomy and removal of the heart, atria and ventricles were dissected and weighed separately. Heart, lung and liver fragments were weighed while wet and again after 72 h of drying at 65 °C; these measurements were used to calculate wet-to-dry weight ratios.

### Myocardial histology

Formalin-fixed LV specimens were embedded in paraffin. Hematoxylin and eosin staining was used for myocyte morphometry. In at least 50 cells from each LV, diameter was defined as the shortest cell-border-to-cell-border distance passing through the nucleus [52]. Collagen was assessed separately in Sirius red F3BA-stained sections. Twenty microscopic fields selected at random were evaluated to determine the interstitial collagen fraction [53]. A blind examiner analyzed images acquired at 40 × magnification with a Leica microscope and digital camera connected to a computer running Image-Pro Plus 3.0 (Media Cybernetics, Silver Spring, MD, USA).

### Serum markers of oxidative damage

Serum malondialdehyde (MDA) and protein carbonyl content were measured as markers of lipid and protein oxidation, respectively, with both results normalized to total protein. For the MDA assay [54], serum was combined with 0.67% thiobarbituric acid (TBA) at a 1:1 ratio and heated for 45 min in a 100 °C water bath. Absorbance of the resulting MDA–TBA adduct, with a 1:2 stoichiometry, was read at 535 nm on a Spectra Max 190 microplate reader (Molecular Devices, Sunnyvale, CA, USA). Concentrations were derived using an extinction coefficient of 1.56 × 10^5^ M⁻^1^ cm⁻^1^ and expressed as nmol/mg protein.

Protein carbonyls were quantified spectrophotometrically after reaction with 2,4-dinitrophenylhydrazine (DNPH), following the assay described in reference [55]. Results were expressed as nmol DNPH/mg protein.

### Myocardial antioxidant activity and lipid hydroperoxide concentration

Approximately 100 mg of LV myocardium were processed in 1 mL of chilled phosphate buffer (0.1 M, pH 7.0). After homogenization, samples underwent centrifugation for 15 min at 10,000 × g and 4 °C. The recovered supernatant was used for spectrophotometric measurements of total protein, lipid hydroperoxides, and antioxidant enzyme activities [56]. The enzymes assessed were catalase (CAT; EC 1.11.1.6), superoxide dismutase (SOD; EC 1.15.1.1), and glutathione peroxidase (EC 1.11.1.9).

### Enzymes involved in myocardial energy metabolism

LV fragments weighing approximately 30 mg were homogenized with 0.5-mm zirconium beads in a Bullet Blender (Next Advance, Inc., NY, USA). Processing lasted 5 min at 4 °C. The extraction medium contained Tris–HCl (50 mM, pH 7.4), EDTA (1 mM), Triton X-100 (0.1%), and a protease inhibitor cocktail. Samples were then centrifuged for 15 min at 12,000 rpm and 4 °C. Enzyme assays used the supernatants, and protein content was determined by the Bradford method. Maximal activities were measured for hexokinase (HK; EC 2.7.1.1), phosphofructokinase (PFK; EC 2.7.1.11), pyruvate kinase (PK; EC 2.7.1.40), citrate synthase (CS; EC 4.1.3.7), and β-hydroxyacyl-CoA dehydrogenase (BHADH; EC 1.1.1.35). Each assay was run in triplicate at 25 °C. A Synergy HT microplate reader (BioTek, USA) recorded absorbance every 30 s over 10 min, with the corresponding reaction medium lacking sample serving as the blank. Closely corresponding assay conditions have been published by Rodrigues et al. [57]. The individual reaction conditions used here are specified below.

For HK [58], glucose addition initiated the reaction, and absorbance was monitored at 340 nm. The medium comprised Tris–HCl (75 mM, pH 7.2), MgCl₂ (7.5 mM), EDTA (0.8 mM), KCl (1.5 mM), 2-mercaptoethanol (4 mM), NADP⁺ (0.4 mM), ATP (2.5 mM), glucose (1 mM), glucose-6-phosphate dehydrogenase (1.4 U), and Triton X-100 (0.05%). For PFK [59], recordings at 340 nm began after addition of fructose-6-phosphate. At pH 8.2, the assay contained Tris–HCl (50 mM), MgSO₄ (2 mM), KCl (5 mM), NADH (0.2 mM), ATP (1 mM), fructose-6-phosphate (3 mM), phosphoenolpyruvate (PEP; 2 mM), lactate dehydrogenase (2 U), pyruvate kinase (4 U), and Triton X-100 (0.05%). For PK [60], PEP was the initiating substrate and 340 nm was the measurement wavelength. The reaction medium contained potassium phosphate (38 mM, pH 7.4), PEP (0.43 mM), β-NAD (0.2 mM; AQ5), magnesium sulfate (6.7 mM), adenosine 5′-diphosphate (1.3 mM), lactate dehydrogenase (20 U), fructose 1,6-bisphosphate (1 mM), and Triton X-100 (0.05%). For CS [61], oxaloacetate addition initiated a reaction monitored at 412 nm. Assay constituents were Tris–HCl (50 mM, pH 8.2), EDTA (1 mM), DTNB (0.2 mM), acetyl-CoA (0.1 mM), oxaloacetate (0.5 mM), and Triton X-100 (0.05%). For BHADH, absorbance at 340 nm was measured after acetoacetyl-CoA addition. Potassium phosphate (100 mM), NADH (0.45 mM), and acetoacetyl-CoA (0.1 mM) constituted the reaction medium.

### Myocardial matrix metalloproteinase activity

Zymography was used to assess myocardial matrix metalloproteinase activity [62]. Cardiac specimens (35 mg) were macerated in extraction buffer and centrifuged. Supernatant protein concentrations were measured by the Bradford assay. Three MMP-2 bands were evaluated according to their position in the gel: upper, intermediate, and lower. For normalization, each gel included a sample from a control animal against which its MMP-2 bands were compared.

### Myocardial transcriptome analysis

#### Library preparation and sequencing

Next-generation sequencing (NGS) was used to compare myocardial transcriptional profile between AS and Sham and between AS-Emp and AS. Four rats per group were included; the experimental procedures were performed in triplicate. LV fragments were taken from the same anatomical region for RNA extraction and purification with the RNeasy kit (Qiagen, Valencia, USA); the workflow also used a QIAcube Connect instrument (Qiagen). RNA quality was assessed on Agilent TapeStation equipment (Agilent Technologies, Santa Clara, EUA) using the RNA Screen Tape Analysis kit. Every sample had an RNA integrity number (RIN) above 9.0. Poly(a)-enriched cDNA libraries were generated using the Illumina Stranded mRNA Prep kit (Ilumina) according to the manufacturer’s instructions. Library quality and quantity were assessed with High Sensitivity DNA Screen Tape analysis on the TapeStation equipment. Sequencing was carried out using the NextSeq 2000 equipment, and a P3 reagents kit with 200 cycles.

#### Bioinformatic analysis

Expression differences for the two comparisons were analyzed in R with DESeq2 package. Genes with an adjusted p-value < 0.05 and a log2 fold change (log2FC) > 1 or < −1 were classified as differentially expressed genes (DEGs), corresponding to upregulated and downregulated genes, respectively. Gene annotations were performed using AnnotationDBI and Org.Rn.eg.db to facilitate the annotation of gene symbols and identifiers. Functional interpretation of the differentially expressed genes used Gene Ontology (GO) [63] enrichment through Enrichr [64]. The enrichment analysis allowed the identification of biological identities associated with DEGs. GOs terms with FDR < 0.01 were considered significant.

### Statistical analysis

Parametric variables are summarized as means ± standard deviations; non-parametric variables are reported as medians with percentiles. Variables were compared by analysis of variance (ANOVA) for a 2 × 2 factorial design followed by Tukey's test or Kruskal–Wallis and Dunn`s tests. Goodman’s test was applied to compare mortality and the frequencies of heart failure features. The significance threshold was *p* < 0.05.

## Data Availability

The data generated in this study are available from the corresponding author upon reasonable request. Transcriptomic data are publicly available in the NCBI Gene Expression Omnibus (GEO) repository under accession number GSE 347984.

## References

[1] Généreux P, Sharma RP, Cubeddu RJ, Aaron L, Abdelfattah OM, Koulogiannis KP, et al. The mortality burden of untreated aortic stenosis. J Am Coll Cardiol. 2023;82(22):2101–9. 10.1016/j.jacc.2023.09.796. 37877909

[2] Palaniappan LP, Allen NB, Almarzooq ZI, Anderson CAM, Arora P, Avery CL, American Heart Association Council on Epidemiology and Prevention Statistics Committee and Stroke Statistics Committee, et al. 2026 heart disease and stroke statistics: A report of US and global data from the American Heart Association. Circulation. 2026;153:e275–906. 10.1161/CIR.0000000000001412. Erratum in: Circulation. 2026;154:e231-2. 10.1161/CIR.0000000000001469.

[3] Grbac AJ, Lee MGY, Chye D, Zhou JY, Batchelor RJ, Steinfort MLE, et al. Management of asymptomatic severe aortic stenosis: A critical review of guidelines and clinical outcomes. Am Heart J. 2025;288:28–40. 10.1016/j.ahj.2025.04.012. 40246047

[4] Cohn JN, Ferrari R, Sharpe N. Cardiac remodeling—concepts and clinical implications: A consensus paper from an international forum on cardiac remodeling. J Am Coll Cardiol. 2000;35(3):569–82. 10.1016/s0735-1097(99)00630-0. 10716457

[5] Reyes DRA, Gomes MJ, Rosa CM, Pagan LU, Zanati SG, Damatto RL, et al. Exercise during transition from compensated left ventricular hypertrophy to heart failure in aortic stenosis rats. J Cell Mol Med. 2019;23(2):1235–45. 10.1111/jcmm.14025. PMC6349163 30456799

[6] Briasoulis A, Androulakis E, Christophides T, Tousoulis D. The role of inflammation and cell death in the pathogenesis, progression and treatment of heart failure. Heart Fail Rev. 2016;21(2):169–76. 10.1007/s10741-016-9533-z. 26872673

[7] Rodrigues EA, Lima ARR, Gomes MJ, Souza LM, Pontes THD, Pagan LU, et al. Influence of isolated resistance exercise on cardiac remodeling, myocardial oxidative stress, and metabolism in infarcted rats. Antioxidants. 2023;12(4):896. 10.3390/antiox12040896. PMC10135620 37107271

[8] Herwig M, Sieme M, Kovács A, Khan M, Mügge A, Schmidt WE, et al. Diabetes mellitus aggravates myocardial inflammation and oxidative stress in aortic stenosis: a mechanistic link to HFpEF features. Cardiovasc Diabetol. 2025;24(1):203. 10.1186/s12933-025-02748-y. PMC12070770 40361188

[9] Otto CM, Nishimura RA, Bonow RO, Carabello BA, Erwin JP, Gentile F, et al. 2020 ACC/AHA guideline for the management of patients with valvular heart disease: Executive summary: A report of the American College of Cardiology/American Heart Association joint committee on clinical practice guidelines. Circulation. 2021;143(5):e35–71. 10.1161/CIR.0000000000000932. 33332149

[10] Vahanian A, Beyersdorf F, Praz F, Milojevic M, Baldus S, Bauersachs J, et al. 2021 ESC/EACTS guidelines for the management of valvular heart disease. Eur Heart J. 2022;43(7):561–632. 10.1093/eurheartj/ehab395. 34453165

[11] Braunwald E. Gliflozins in the management of cardiovascular disease. N Engl J Med. 2022;386(21):2024–34. 10.1056/NEJMra2115011. 35613023

[12] Zinman B, Wanner C, Lachin JM, Fitchett D, Bluhmki E, Hantel S, et al. Empagliflozin, cardiovascular outcomes, and mortality in type 2 diabetes. N Engl J Med. 2015;373(22):2117–28. 10.1056/NEJMoa1504720. 26378978

[13] Braunwald E. SGLT2 inhibitors: the statins of the 21st century. Eur Heart J. 2022;43(11):1029–30. 10.1093/eurheartj/ehab765. 34741610

[14] The EMPA-KIDNEY Collaborative Group. Long-term effects of empagliflozin in patients with chronic kidney disease. N Engl J Med. 2025;392(8):777–87. 10.1056/NEJMoa2409183. PMC7616743 39453837

[15] McDonagh TA, Metra M, Adamo M, Gardner RS, Baumbach A, Böhm M, et al. 2023 focused update of the 2021 ESC guidelines for the diagnosis and treatment of acute and chronic heart failure. Eur Heart J. 2023;44(37):3627–39. 10.1093/eurheartj/ehad195. 37622666

[16] Heidenreich PA, Bozkurt B, Aguilar D, Allen LA, Byun JJ, Colvin MM, et al. 2022 AHA/ACC/HFSA guideline for the management of heart failure: A report of the American College of Cardiology/American Heart Association joint committee on clinical practice guidelines. Circulation. 2022;145(18):e876–94. 10.1161/CIR.0000000000001062. 35363499

[17] Zhang X, Zhou C, Chen G, Li Y, Liu X, Luo S. Dapagliflozin promotes metabolic reprogramming against myocardial infarction through the MAPK-FOXO3-STC1 and HIF-1a-STC1 pathways. Life Sci. 2025;377:123798. 10.1016/j.lfs.2025.123798. 40480620

[18] Rosa CM, Campos DHS, Reyes DRA, Damatto FC, Kurosaki LY, Pagan LU, et al. Effects of the SGLT2 inhibition on cardiac remodeling in streptozotocin-induced diabetic rats, a model of type 1 diabetes mellitus. Antioxidants. 2022;11(5):982. 10.3390/antiox11050982. PMC9137562 35624845

[19] Kolijn D, Pabel S, Tian Y, Lódi M, Herwig M, Carrizzo A, et al. Empagliflozin improves endothelial and cardiomyocyte function in human heart failure with preserved ejection fraction via reduced pro-inflammatory-oxidative pathways and protein kinase Gα oxidation. Cardiovasc Res. 2021;117(2):495–507. 10.1093/cvr/cvaa123. 32396609

[20] Yu H, Han Z, Chang W, Zhou H, Du B, Jia B, et al. Canagliflozin ameliorates myocardial fibrosis and cardiac function in chronic heart failure: A dose-independent therapeutic approach. J Cell Mol Med. 2025;29(15):e70718. 10.1111/jcmm.70718. PMC12335939 40785031

[21] Mylonas N, Nikolaou PE, Siokatas G, Kostopoulos IV, Tsitsilonis O, Drosatos K, et al. Empagliflozin restores cardiac metabolism and suppresses immune activation in acute myocardial infarction. Atherosclerosis. 2025;407:120404. 10.1016/j.atherosclerosis.2025.120404. PMC12311800 40543297

[22] Pabel S, Wagner S, Bollenberg H, Bengel P, Kovács Á, Schach C, et al. Empagliflozin directly improves diastolic function in human heart failure. Eur J Heart Fail. 2018;20(12):1690–700. 10.1002/ejhf.1328. 30328645

[23] Shah T, Zhang Z, Shah H, Fanaroff AC, Nathan AS, Parise H, et al. Effect of sodium-glucose cotransporter-2 inhibitors on the progression of aortic stenosis. JACC Cardiovasc Interv. 2025;18(6):738–48. 10.1016/j.jcin.2024.11.036. 39985508

[24] Okoshi MP, Cezar MDM, Iyomasa RM, Silva MB, Costa LCO, Martinez PF, et al. Effects of early aldosterone antagonism on cardiac remodeling in rats with aortic stenosis-induced pressure overload. Int J Cardiol. 2016;222:569–75. 10.1016/j.ijcard.2016.07.266. 27513653

[25] Byrne NJ, Parajuli N, Levasseur JL, Boisvenue J, Beker DL, Masson G, et al. Empagliflozin prevents worsening of cardiac function in an experimental model of pressure overload-induced heart failure. JACC Basic Transl Sci. 2017;2(4):347–54. 10.1016/j.jacbts.2017.07.003. PMC6034464 30062155

[26] Verma S, Garg A, Yan AT, Gupta AK, Al-Omran M, Sabongui A, et al. Effect of empagliflozin on left ventricular mass and diastolic function in individuals with diabetes: an important clue to the EMPA-REG OUTCOME trial? Diabetes Care. 2016;39(12):e212–3. 10.2337/dc16-1312. 27679584

[27] Marwick TH, Halabi A, Soh CH, Curtin A, Sherrif AG, Azad A, et al. Effects of dapagliflozin on the progression of left ventricular dysfunction in type 2 diabetes mellitus: a randomized controlled trial. Cardiovasc Diabetol. 2025;24(1):232. 10.1186/s12933-025-02796-4. PMC12125862 40448135

[28] Abou Warda AE, Duarte JD, Nabil Salem M, Salem HF, Moharram AN, Alanazi AS, et al. Dapagliflozin enhances systolic and diastolic function in concordance with clinical response in patients with left ventricular dysfunction. Eur Rev Med Pharmacol Sci. 2025;29(5):231–47. 10.26355/eurrev_202505_37222. 40444607

[29] Packer M. SGLT2 inhibitors: role in protective reprogramming of cardiac nutrient transport and metabolism. Nat Rev Cardiol. 2023;20(7):443–62. 10.1038/s41569-022-00824-4. 36609604

[30] Packer M, Wilcox CS, Testani JM. Critical Analysis of the Effects of SGLT2 inhibitors on renal tubular sodium, water and chloride homeostasis and their role in influencing heart failure outcomes. Circulation. 2023;148(4):354–72. 10.1161/CIRCULATIONAHA.123.064346. PMC10358443 37486998

[31] Marfella R, Scisciola L, D’Onofrio N, Maiello C, Trotta MC, Sardu C, et al. Sodium-glucose cotransporter-2 (SGLT2) expression in diabetic and non-diabetic failing human cardiomyocytes. Pharmacol Res. 2022;184:106448. 10.1016/j.phrs.2022.106448. 36096423

[32] Scisciola L, Paolisso P, Belmonte M, Gallinoro E, Delrue L, Taktaz F, et al. Myocardial sodium–glucose cotransporter 2 expression and cardiac remodelling in patients with severe aortic stenosis: The BIO-AS study. Eur J Heart Fail. 2024;26(2):471–82. 10.1002/ejhf.3145. 38247224

[33] Roman-Pepine D, Serban AM, Capras RD, Cismaru CM, Filip AG. A comprehensive review: Unraveling the role of inflammation in the etiology of heart failure. Heart Fail Rev. 2025;30(5):931–54. 10.1007/s10741-025-10519-w. PMC12297024 40360833

[34] Reyes DRA, Gomes MJ, Rosa CM, Pagan LU, Damatto FC, Damatto RL, et al. N-acetylcysteine influence on oxidative stress and cardiac remodeling in rats during transition from compensated left ventricular hypertrophy to heart failure. Cell Physiol Biochem. 2017;44(6):2310–21. 10.1159/000486115. 29258061

[35] Berk BC, Fujiwara K, Lehoux S. ECM remodeling in hypertensive heart disease. J Clin Invest. 2007;117(3):568–75. 10.1172/JCI31044. PMC1804378 17332884

[36] Karakasis P, Theofilis P, Vlachakis PK, Apostolos A, Milaras N, Ktenopoulos N, et al. SGLT2 inhibitors and cardiac fibrosis: a comprehensive review. Curr Probl Cardiol. 2025;50(10):103149. 10.1016/j.cpcardiol.2025.103149. 40769233

[37] Chase D, Eykyn TR, Shattock MJ, Chung YJ. Empagliflozin improves cardiac energetics during ischaemia/reperfusion by directly increasing cardiac ketone utilization. Cardiovasc Res. 2023;119(16):2672–80. 10.1093/cvr/cvad157. PMC10730240 37819017

[38] Suzuki Y, Amano T. Revisiting the thrifty substrate hypothesis: ketone bodies as cardioprotective fuels in diabetes and SGLT2 inhibitor therapy. J Diabetes Investig. 2025;16(7):1154–6. 10.1111/jdi.70068. PMC12209502 40331922

[39] Alzhrani AA, Rasool M, Karim S, Alhejin A, Haque A, Morsi M, et al. KDM3A knockdown regulates COMP, LOX, COL8A1 and ACOT1 genes in myocardial fibrosis. Bioinformation. 2024;20(4):305–13. 10.6026/973206300200305. PMC11161882 38854759

[40] Saadat S, Noureddini M, Mahjoubin-Tehran M, Nazemi S, Shojaie L, Aschner M, et al. Pivotal role of TGF-β/Smad signaling in cardiac fibrosis: non-coding RNAs as effectual players. Front Cardiovasc Med. 2021;7:588347. 10.3389/fcvm.2020.588347. PMC7868343 33569393

[41] Chen XZ, Li XM, Xu SJ, Hu S, Wang T, Li RF, et al. TMEM11 regulates cardiomyocyte proliferation and cardiac repair via METTL1-mediated m^7^G methylation of ATF5 mRNA. Cell Death Differ. 2023;30(7):1786–98. 10.1038/s41418-023-01179-0. PMC10307882 37286744

[42] Bilchick KC, Saha SK, Mikolajczyk E, Cope L, Ferguson WJ, Yu W, et al. Differential regional gene expression from cardiac dyssynchrony induced by chronic right ventricular free wall pacing in the mouse. Physiol Genomics. 2006;26(2):109–15. 10.1152/physiolgenomics.00281.2005. 16670254

[43] Gabrielsen A, Lawler PR, Yongzhong W, Steinbrüchel D, Blagoja D, Paulsson-Berne G, et al. Gene expression signals involved in ischemic injury, extracellular matrix composition and fibrosis defined by global mRNA profiling of the human left ventricular myocardium. J Mol Cell Cardiol. 2007;42(4):870–83. 10.1016/j.yjmcc.2006.12.016. 17343875

[44] Awasthi S, Hindle A, Sawant NA, George M, Vijayan M, Kshirsagar S, et al. RALBP1 in oxidative stress and mitochondrial dysfunction in Alzheimer’s disease. Cells. 2021;10(11):3113. 10.3390/cells10113113. PMC8620796 34831336

[45] Simpson KE, Staikos CA, Watson KL, Moorehead RA. Loss of MXRA8 delays mammary tumor development and impairs metastasis. Int J Mol Sci. 2023;24(18):13730. 10.3390/ijms241813730. PMC10530983 37762032

[46] Vogel C, Marcotte EM. Insights into the regulation of protein abundance from proteomic and transcriptomic analyses. Nat Rev Genet. 2012;13(4):227–32. 10.1038/nrg3185. PMC3654667 22411467

[47] Baka T, Moore J, Qin F, Yurista SR, Zhang A, He H, et al. Empagliflozin enhances metabolic efficiency and improves left ventricular hypertrophy in a hypertrophic cardiomyopathy mouse model. Eur Heart J. 2025;46(40):4105–19. 10.1093/eurheartj/ehaf324. PMC12539926 40396194

[48] Souza LM, Gomes MJ, Brandao BB, Pagan LU, Gatto M, Damatto FC, et al. Effects of resistance exercise on slow-twitch soleus muscle of infarcted rats. Antioxidants. 2023;12(2):291. 10.3390/antiox12020291. PMC9952394 36829850

[49] Cezar MD, Damatto RL, Martinez PF, Lima AR, Campos DH, Rosa CM, et al. Aldosterone blockade reduces mortality without changing cardiac remodeling in spontaneously hypertensive rats. Cell Physiol Biochem. 2013;32(5):1275–87. 10.1159/000354526. 24281393

[50] Zornoff LAM, Matsubara LS, Matsubara BB, Okoshi MP, Okoshi K, Dal Pai-Silva M, et al. Beta-carotene supplementation attenuates cardiac remodeling induced by one-month tobacco-smoke exposure in rats. Toxicol Sci. 2006;90(1):259–66. 10.1093/toxsci/kfj080. 16387745

[51] Pagan LU, Damatto RL, Gomes MJ, Lima ARR, Cezar MDM, Damatto FC, et al. Low-intensity aerobic exercise improves cardiac remodelling of adult spontaneously hypertensive rats. J Cell Mol Med. 2019;23(9):6504–7. 10.1111/jcmm.14530. PMC6714166 31317657

[52] Guizoni DM, Oliveira-Junior SA, Noor SLR, Pagan LU, Martinez PF, Lima ARR, et al. Effects of late exercise on cardiac remodeling and myocardial calcium handling proteins in rats with moderate and large size myocardial infarction. Int J Cardiol. 2016;221:406–12. 10.1016/j.ijcard.2016.07.072. 27404715

[53] Damatto RL, Lima ARR, Martinez PF, Cezar MDM, Okoshi K, Okoshi MP. Myocardial myostatin in spontaneously hypertensive rats with heart failure. Int J Cardiol. 2016;215:384–7. 10.1016/j.ijcard.2016.04.101. 27128567

[54] Uchiyama M, Mihara M. Determination of malonaldehyde precursor in tissues by thiobarbituric acid test. Anal Biochem. 1978;86(1):271–8. 10.1016/0003-2697(78)90342-1. 655387

[55] Mesquita CS, Oliveira R, Bento F, Geraldo D, Rodrigues JV, Marcos JC. Simplified 2,4-dinitrophenylhydrazine spectrophotometric assay for quantification of carbonyls in oxidized proteins. Anal Biochem. 2014;458:69–71. 10.1016/j.ab.2014.04.034. 24814294

[56] Gomes MJ, Pagan LU, Lima ARR, Reyes DRA, Martinez PF, Damatto FC, et al. Effects of aerobic and resistance exercise on cardiac remodelling and skeletal muscle oxidative stress of infarcted rats. J Cell Mol Med. 2020;24(9):5352–62. 10.1111/jcmm.15191. PMC7205792 32239667

[57] Rodrigues EA, Rosa CM, Campos DHS, Damatto FC, Murata GM, Souza LM, et al. The influence of dapagliflozin on cardiac remodeling, myocardial function and metabolomics in type 1 diabetes mellitus rats. Diabetol Metab Syndr. 2023;15(1):223. 10.1186/s13098-023-01196-6. PMC10617150 37908006

[58] Crabtree B, Newsholme EA. The activities of phosphorylase, hexokinase, phosphofructokinase, lactate dehydrogenase and the glycerol 3-phosphate dehydrogenase in muscles from vertebrates and invertebrates. Biochem J. 1972;126(1):49–58. 10.1042/bj1260049. PMC1178350 4342385

[59] Hengartner H, Harris JI. Purification by affinity chromatography, properties and crystallisation of phosphofructokinase from thermophilic micro-organisms. FEBS Lett. 1975;55(1–2):282–5. 10.1016/0014-5793(75)81012-x. 124670

[60] Cardenas JM, Dyson RD, Strandholm JJ, Bovine pyruvate kinases I. Purification and characterization of the skeletal muscle isozyme. J Biol Chem. 1973;248(20):6931–7. 10.1016/S0021-9258(19)43342-5. 4200582

[61] Alp PR, Newsholme EA, Zammit VA. Activities of citrate synthase and NAD+-linked and NADP+-linked isocitrate dehydrogenase in muscle from vertebrates and invertebrates. Biochem J. 1976;154(3):689–700. 10.1042/bj1540689. PMC1172771 8036

[62] Tyagi SC, Ratajska A, Weber KT. Myocardial matrix metalloproteinase(s): localization and activation. Mol Cell Biochem. 1993;126(1):49–59. 10.1007/BF01772207. 8107689

[63] Ashburner M, Ball CA, Blake JA, Botstein D, Butler H, Cherry JM, et al. Gene ontology: tool for the unification of biology. Gene Ontology Consortium Nat Genet. 2000;25(1):25–9. 10.1038/75556. PMC3037419 10802651

[64] Kuleshov MV, Jones MR, Rouillard AD, Fernandez NF, Duan Q, Wang Z, et al. Enrichr: a comprehensive gene set enrichment analysis web server 2016 update. Nucleic Acids Res. 2016;44(W1):W90–7. 10.1093/nar/gkw377. PMC4987924 27141961

